# Genotype and Allele Frequencies of Canine Degenerative Myelopathy‐Associated *SOD1* Gene Variant in Irish Wolfhound Dogs

**DOI:** 10.1002/age.70111

**Published:** 2026-05-05

**Authors:** Yoshihiko Yu, Margret L. Casal

**Affiliations:** ^1^ Department of Clinical Sciences and Advanced Medicine, School of Veterinary Medicine University of Pennsylvania Philadelphia Pennsylvania USA

**Keywords:** canine, *Canis lupus familiaris*, frequency, genetic epidemiology, genetic test, genotyping, superoxide dismutase 1

Canine degenerative myelopathy (DM) is a slowly progressive, late‐onset neurodegenerative disorder presenting with thoracolumbar myelopathy, which may eventually progress to various impairments, such as respiratory dysfunctions, leading to death (Coates and Wininger 2010; Kobatake et al. 2021). Dogs homozygous for a single nucleotide variant in *SOD1* (NM_001003035.1:c.118G>A [p.E40K]; NC_006613.4:g.26532306G>A) are considered to be at high risk for DM, and this variant has been reported to be prevalent in many canine breeds (Awano et al. 2009; Zeng et al. 2014). Heterozygous dogs rarely develop the disease (Zeng et al. 2014). The definitive diagnosis is only made by histopathology; therefore, antemortem presumptive diagnosis is made based on the combination of clinical findings, including lack of clinically relevant neuroimaging findings, and detection of the homozygous *SOD1* variant (Coates and Wininger 2010). Several diseases may mimic DM or coexist with it, including degenerative lumbosacral syndrome, intervertebral disc disease, spinal cord neoplasia, and some orthopedic disorders (Kneller et al. 1975). Thus, genetic testing is important for antemortem diagnosis (Bouché et al. 2023).

The Irish Wolfhound (IW) is a large breed in which hindlimb dysfunction markedly impacts the quality of life for both dogs and their owners and may ultimately lead to euthanasia. No histopathologically confirmed DM cases have been reported in this breed; however, some owners and veterinarians have expressed anecdotal concern about DM in IW dogs, despite the lack of evidence.

It remains uncertain whether the *SOD1* variant is prevalent in IW dogs and whether DM should be routinely considered in the differential diagnosis of aged IW dogs with thoracolumbar myelopathy. A previous study identified three heterozygous and 40 wild‐type dogs out of 43 genotyped (Zeng et al. 2014); however, further studies involving a larger number of dogs are needed to accurately determine genotype and allele frequencies. Therefore, this study aimed to determine the frequencies of the *SOD1* c.118G>A genotype and allele in a larger population of IW dogs born in North America during the recent decade (2014–2023).

Genotyping of the single nucleotide variant was conducted using a custom TaqMan SNP genotyping assay (Thermo Fisher Scientific, Waltham, MA, USA), with the primer and probe sequences previously reported (Chang et al. 2013). To minimize overestimation, only one littermate was genotyped when multiple full siblings were available. The detailed methods are described in Supporting Information.

In our IW population, 262 dogs were wild‐type (98.1%: 95% confidence interval [CI], 95.7%–99.2%), five dogs were heterozygous (1.9%: 95% CI, 0.8%–4.3%), and none were homozygous (0%: 95% CI, 0%–1.4%), resulting in a minor allele frequency of 0.009 (95% CI, 0.004–0.022). These frequencies were lower than those reported previously in this breed and in many other dog breeds (Zeng et al. 2014; Donner et al. 2018) and suggest that the occurrence of DM specifically associated with the common variant in *SOD1* (c.118G>A) appears unlikely when considering a differential diagnosis in aged IW dogs presenting with thoracolumbar myelopathy in the North American population. Additional information, including results and comments, is also provided in the Supporting Information.

## Author Contributions

**Yoshihiko Yu:** conceptualization, data curation, methodology, investigation, formal analysis, writing – original draft, writing – review and editing, visualization. **Margret L. Casal:** conceptualization, data curation, resources, funding acquisition, supervision, writing – review and editing, validation.

## Funding

This study was supported by The Irish Wolfhound Foundation. The funders had no role in study design, data collection and analysis, decision to publish, or preparation of the manuscript. Open access funding was provided by the University of Pennsylvania.

## Ethics Statement

Dog samples used in this study were archival and were collected as owner donations with written informed consent.

## Conflicts of Interest

One of the authors (M.L.C.) operates a laboratory (PennGen Laboratories, School of Veterinary Medicine, University of Pennsylvania) that offers both fee‐for‐service and direct‐to‐consumer genetic testing for the canine *SOD1* variant (c.118G>A).

## Supporting information


**Table S1:** The primer and probe sequences used for genotyping in this study.
**Table S2:** The primer sequences used for conventional PCR and Sanger sequencing in this study.
**Table S3:** The distribution of birth years of all the genotyped dogs and heterozygous dogs.
**Figure S1:** A representative allelic discrimination plot from real‐time PCR‐based SNV genotyping using TaqMan probes for canine degenerative myelopathy‐associated SOD1 variant (118G>A). Each dot represents a single reaction well. Distinct clustering of red dots, light green dots, and blue dots indicates wild‐type (G/G), heterozygous (G/A), and homozygous (A/A) genotypes, respectively. No‐template controls show no detectable amplification. Positive and negative controls are included in triplicate. Cross marks indicate undetermined calls.
**Figure S2:** Representative amplification plots and multicomponent plots from real‐time PCR‐based SNV genotyping using TaqMan probes for canine degenerative myelopathy‐associated SOD1 variant (118G>A). (A, C, E) Amplification plots showing successful amplification of allele‐specific fluorescence signals, with the wild‐type allele indicated in light green and the variant allele in blue: (A) wild‐type (G/G), (C) heterozygous (G/A), (E) homozygous (A/A). (B, D, F) Corresponding multicomponent plots from the same reaction wells, demonstrating appropriate VIC and FAM signal behavior and a stable ROX passive reference signal, indicated in light green, blue, and red, respectively: (B) wild‐type (G/G), (D) heterozygous (G/A), (F) homozygous (A/A). These plots were visually inspected as a quality control measure to support interpretation of genotyping results.
**Data S1:** Individual‐level genotype data of the study population. This supplementary dataset includes the following variables for each dog: Laboratory ID number (Lab ID#), genotype, birth year, sex, and cause of death. Sex is coded as 1 = male and 2 = female. Cause of death reflects the information available at the time this study was conducted.

**Data S1:** age70111‐sup‐0002‐DataS1.xlsx.

## Data Availability

All data generated or analyzed during this study are included in this article and its Supporting Information files.
