# Supplementary material for: Genotype and Allele Frequencies of Canine Degenerative Myelopathy‐Associated SOD1 Gene Variant in Irish Wolfhound Dogs
Source: Anim Genet. 2026 May 5;57:e70111. doi: 10.1002/age.70111 (PMC13139880; doi:10.1002/age.70111)
Supplement: Supplementary file 1 — Table S1: The primer and probe sequences used for genotyping in this study. Table S2: The primer sequences used for conventional PCR and Sanger sequencing in this study. Table S3: The distribution of birth years of all the genotyped dogs and heterozygous dogs. Figure S1: A representative allelic discrimination plot from real‐time PCR‐based SNV genotyping using TaqMan probes for canine degenerative myelopathy‐associated SOD1 variant (118G>A). Each dot represents a single reaction well. Distinct clustering of red dots, light green dots, and blue dots indicates wild‐type (G/G), heterozygous (G/A), and homozygous (A/A) genotypes, respectively. No‐template controls show no detectable amplification. Positive and negative controls are included in triplicate. Cross marks indicate undetermined calls. Figure S2: Representative amplification plots and multicomponent plots from real‐time PCR‐based SNV genotyping using TaqMan probes for canine degenerative myelopathy‐associated SOD1 variant (118G>A). (A, C, E) Amplification plots showing successful amplification of allele‐specific fluorescence signals, with the wild‐type allele indicated in light green and the variant allele in blue: (A) wild‐type (G/G), (C) heterozygous (G/A), (E) homozygous (A/A). (B, D, F) Corresponding multicomponent plots from the same reaction wells, demonstrating appropriate VIC and FAM signal behavior and a stable ROX passive reference signal, indicated in light green, blue, and red, respectively: (B) wild‐type (G/G), (D) heterozygous (G/A), (F) homozygous (A/A). These plots were visually inspected as a quality control measure to support interpretation of genotyping results. Data S1: Individual‐level genotype data of the study population. This supplementary dataset includes the following variables for each dog: Laboratory ID number (Lab ID#), genotype, birth year, sex, and cause of death. Sex is coded as 1 = male and 2 = female. Cause of death reflects the information available at the time this study was [file AGE-57-0-s001.pdf]

Supporting Information

Genotype and allele frequencies of canine degenerative myelopathy-associated *SOD1* gene variant in Irish Wolfhound dogs

Yoshihiko Yu[1], Margret L Casal[1]

[1] Department of Clinical Sciences and Advanced Medicine, School of Veterinary Medicine, University of Pennsylvania, Philadelphia, PA, USA.

Supplementary Materials and Methods

For the genetic studies of Irish Wolfhound (IW) dogs, whole blood samples were submitted to the Section of Medical Genetics, Department of Clinical Sciences and Advanced Medicine, School of Veterinary Medicine, University of Pennsylvania (Philadelphia, PA, USA) with informed consent forms completed by owners. Genomic DNA was extracted from whole blood using a modified organic extraction method or a commercially available column-based DNA extraction kit.

The DNA archive collected from IW dogs included many related individuals, including full-sibling pairs. If many related individuals are included, certain genotypes may be overrepresented, potentially leading to an overestimation of their true frequency in the population. Therefore, samples from our IW DNA archive were included for genotyping if they met the following criteria: (1) the dog was registered with either the American Kennel Club (AKC) or the Canadian Kennel Club (CKC), and thus possessing an official registration number; (2) when samples from the same individual had been provided to the DNA archive multiple times, only one representative sample was used to avoid duplication; (3) when multiple full siblings were available, only one dog per litter was selected for genotyping to minimize overestimation of allele frequency; and (4) when issues such as low DNA concentration, DNA degradation, or genotyping failure due to these issues were identified, an alternative sample from a full sibling was used whenever available. Furthermore, dogs with failed genotyping were excluded from subsequent statistical analyses. Using data extracted from our database, data cleaning and case selection were performed for AKC-registered dogs using R (version 4.5.1; R Core Team, 2025) with the dplyr package, including grouping dogs by litter identifier and selecting a single representative per litter when multiple littermates were present. However, this procedure did not apply to CKC-registered dogs, and their information was verified individually.

Genotyping of the *SOD1* gene variant (NM_001003035.1:c.118G>A [p.E40K]; NC_006613.4:g.26532306G>A) was conducted using a real-time PCR instrument (StepOnePlus

Real-Time PCR System; Thermo Fisher Scientific, Waltham, MA, USA) with a custom TaqMan SNP genotyping assay (Thermo Fisher Scientific) and TaqPath ProAmp Master Mix (Thermo Fisher Scientific). The primer and probe sequences used in this study were identical to those previously reported (Chang et al., 2013), and are listed in **Supplementary Table 1**. This primer and probe set was deemed not to be interfered with by previously reported insertion or deletion variants located near the region of interest and found in other canine breeds (Turba et al., 2017; Santos et al., 2020), and thus was considered suitable for genotyping. The real-time PCR amplifications were performed in a final volume of 10 µl, and the following thermal cycling conditions were used: initial denaturation at 95°C for 10 minutes, followed by 40 cycles at 95°C for 15 seconds and 60°C for 1 minute. Fluorescence was measured at 60 °C for 30 seconds before and after PCR amplification (pre- and post-PCR reads) for allelic discrimination analysis. DNA samples from other dog breeds with wild-type, heterozygous, and homozygous genotypes were used as controls; their genotypes had been previously confirmed by conventional PCR followed by Sanger sequencing using the protocol described below. No-template control reactions were also performed. Genotypes were assigned using the allelic discrimination algorithm implemented in the StepOnePlus software, and the results were visualized on allelic discrimination plots **(Supplementary Figure 1)**. Interpretation of the genotyping results was supported by visual inspection of the amplification plots and multicomponent plots **(Supplementary Figure 2)**.

To validate the real-time PCR-based single nucleotide variant (SNV) genotyping results in the present study, conventional PCR followed by Sanger sequencing was performed for all five heterozygous IW dogs and five randomly selected wild-type IW dogs. Conventional PCR was performed using the Invitrogen Platinum Taq DNA Polymerase (Thermo Fisher Scientific), according to the manufacturer's instructions, with an annealing temperature of 54°C and 35 PCR cycles. The primer sequences used for conventional PCR are provided in **Supplementary Table 2**. PCR amplicons were visualized by agarose gel electrophoresis, and the PCR products were extracted and purified using the QIAquick Gel Extraction Kit (Qiagen, Hilden, Germany). Purified PCR products were submitted to GENEWIZ (Azenta Life Sciences, South Plainfield, NJ, USA), and Sanger sequencing was performed using an Applied Biosystems 3730xl DNA Analyzer (Thermo Fisher Scientific). Sequencing primers are also listed in **Supplementary Table 2**.

Based on real-time PCR-based SNV genotyping result, genotype and allele frequencies were determined. The 95% confidence intervals (95% CIs) were calculated using the Wilson method in R. In addition, sex distribution and birth year distribution were examined; the latter was specifically assessed to evaluate potential temporal sampling bias and cohort effects. When a case carrying the variant allele was identified, information on the dog, including sex, year of birth, pedigree, and cause of death, if available, was obtained from The Irish Wolfhound Database.

Supplementary Results

A total of 267 dogs were successfully genotyped, most of which were AKC-registered dogs. Of these, 109 were male and 158 were female. The five heterozygous dogs consisted of two males and three females. The distribution of birth years of all the genotyped dogs and heterozygous dogs is summarized in **Supplementary Table 3**, demonstrating that heterozygous dogs were not restricted to a single birth cohort. Pedigree analysis indicated that two of the five dogs had a sire-offspring relationship. The cause of death was reviewed when reported; however, information was available for only one heterozygous dog, and the cause was not related to hindlimb dysfunction.

In all dogs subjected to Sanger sequencing, genotypes were fully concordant with real-time PCR results. Additionally, although beyond the scope of this study, a previously reported intronic SNV described in other dog breeds (NC_006613.4:g.26532211del, also previously reported as ENSCAFG00000008859:g.26540247del) (Santos et al., 2020; Kountourantzis et al., 2023) was identified by Sanger sequencing in one dog each from the heterozygous and wild-type genotype groups (2 of 10 dogs). Furthermore, none of the dogs carried a previously reported intronic 54-bp insertion variant (c.166+9_c.166+10ins [KX017978.1:g237_290]) identified in other dog breeds (Turba et al., 2017).

Supplementary Comments

In summary, this study provides information on genotype and allele frequencies of canine degenerative myelopathy-associated *SOD1* gene variant (NM_001003035.1:c.118G>A [p.E40K]; NC_006613.4:g.26532306G>A) in IW dogs in North America. In a previous report, genotyping of 43 IW dogs identified three heterozygous and 40 wild-type dogs (Zeng et al., 2014). In this study, only one dog per litter was included when multiple full siblings were available to avoid overestimation of genotype and allele frequencies. This may partly account for the differences in these frequencies compared with the previous study (Zeng et al., 2014). Moreover, because the previous study (Zeng et al., 2014) was published in 2014 and the present study included individuals born between 2014 and 2023, changes in genotype and allele frequencies may have occurred due to the increasing availability of genetic testing for this variant. Our results indicate that, excluding circumstances such as inbreeding within the lineage carrying the variant, the likelihood of homozygous individuals being born under random mating conditions is low. Indeed, no homozygous dogs were identified among the 267 genotyped individuals in the present study.

Limitations include cryptic relatedness in our samples, reducing the effective sample size; the moderate sample size; and the lack of genotyping for the other variant, which has been reported only in Bernese Mountain Dogs (Wininger et al., 2011; Zeng et al., 2014).

Supplementary Table 1. The primer and probe sequences used for genotyping in this study.

Primer/probe	Sequences (5'–3')	Reporter Dye
Forward Primer Sequence	TGGGCCTGTTGTGGTATCAG	–
Reverse Primer Sequence	CAAACTGATGGACGTGGAATCC	–
Wild-type Probe Sequence	CTCGCCTTCAGTCAGC	VIC
Variant Probe Sequence	CTCGCCTTTAGTCAGC	FAM

Supplementary Table 2. The primer sequences used for conventional PCR and Sanger sequencing in this study.

Primer/probe	Sequences (5'–3')
Forward Primer Sequence	GTCCCCAGCCTAGAATGGTTAA
Reverse Primer Sequence	CGGCTTTGTGGATCATTTCC
Reverse Internal Primer Sequence for Sequencing	TTGAGGATTTTCAATGTTTAGGAGTAGC

The forward primer was used for both PCR and Sanger sequencing, whereas the remaining primers were used only for PCR or Sanger sequencing.

Supplementary Table 3. The distribution of birth years of all the genotyped dogs and heterozygous dogs.

Birth years	Dogs genotyped	*SOD1* (c.118G>A) genotypes			Allele frequency
		Wild-type	Heterozygous	Homozygous	
2014	50	50	0	0	0
2015	43	41	2	0	0.023
2016	31	31	0	0	0
2017	31	30	1	0	0.016
2018	19	18	1	0	0.026
2019	29	29	0	0	0
2020	23	23	0	0	0
2021	20	20	0	0	0
2022	10	10	0	0	0
2023	11	10	1	0	0.045
Total	267	262	5	0	0.009

Supplementary Figure 1. A representative allelic discrimination plot from real-time PCR-based SNV genotyping using TaqMan probes for canine degenerative myelopathy-associated *SOD1* variant (118G>A). Each dot represents a single reaction well. Distinct clustering of red dots, light green dots, and blue dots indicates wild-type (G/G), heterozygous (G/A), and homozygous (A/A) genotypes, respectively. No-template controls show no detectable amplification. Positive and negative controls are included in triplicate. Cross marks indicate undetermined calls.

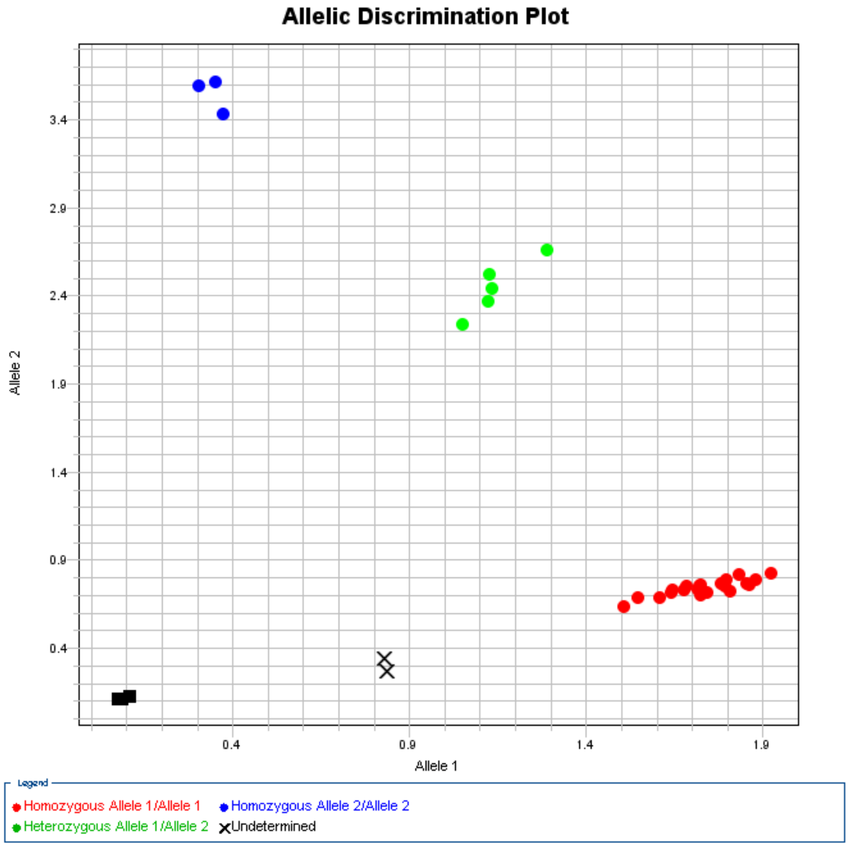

Supplementary Figure 2. Representative amplification plots and multicomponent plots from real-time PCR-based SNV genotyping using TaqMan probes for canine degenerative myelopathy-associated *SOD1* variant (118G>A). **(A, C, E)** Amplification plots showing successful amplification of allele-specific fluorescence signals, with the wild-type allele indicated in light green and the variant allele in blue: (A) wild-type (G/G), (C) heterozygous (G/A), (E) homozygous (A/A). **(B, D, F)** Corresponding multicomponent plots from the same reaction wells, demonstrating appropriate VIC and FAM signal behavior and a stable ROX passive reference signal, indicated in light green, blue, and red, respectively: (B) wild-type (G/G), (D) heterozygous (G/A), (F) homozygous (A/A). These plots were visually inspected as a quality control measure to support interpretation of genotyping results.

(A)

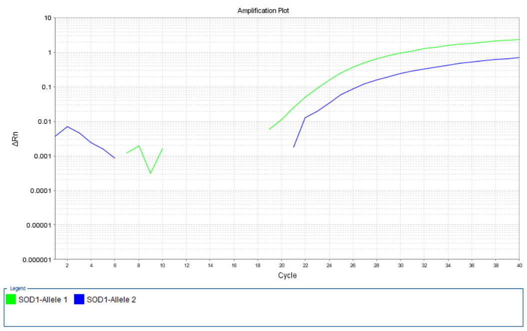
Amplification Plot
ΔRn
Cycle
Legend
SOD1-Allele 1
SOD1-Allele 2

(B)

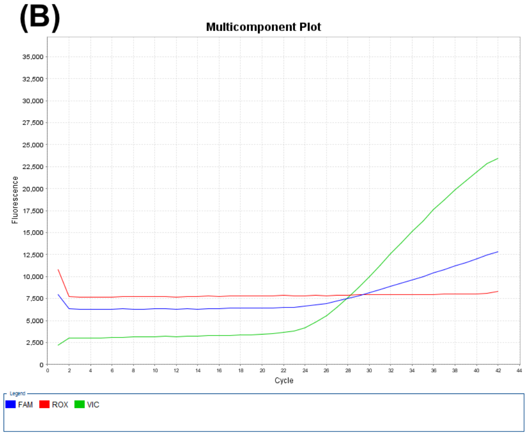
Multicomponent Plot
Fluorescence
Cycle
Legend
FAM
ROX
VIC

(C)

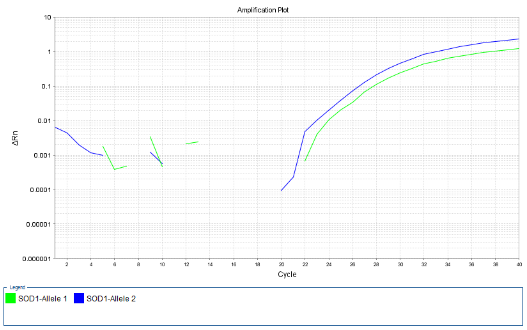
Amplification Plot
ΔRn
Cycle
Legend
SOD1-Allele 1
SOD1-Allele 2

(D)

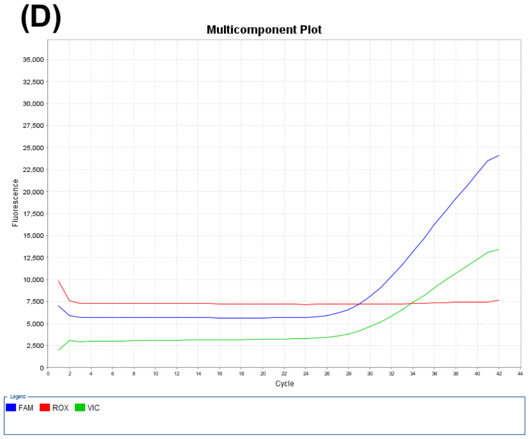
Multicomponent Plot
Fluorescence
Cycle
Legend
FAM
ROX
VIC

(E)

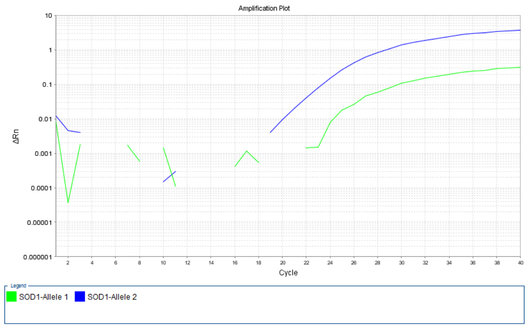
Amplification Plot
ΔRn
Cycle
Legend
SOD1-Allele 1
SOD1-Allele 2

(F)

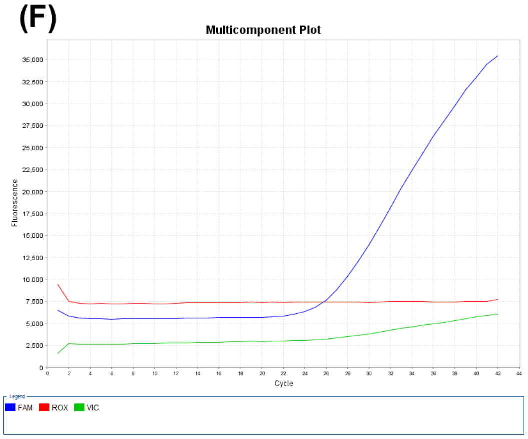
Multicomponent Plot
Fluorescence
Cycle
Legend
FAM
ROX
VIC

Supplementary Data 1. Individual-level genotype data of the study population. This supplementary dataset includes the following variables for each dog: Laboratory ID number (Lab ID#), genotype, birth year, sex, and cause of death. Sex is coded as 1 = male and 2 = female. Cause of death reflects the information available at the time this study was conducted. This Supplementary Data file is provided separately and is available online.

Supplementary References

Chang, H.-S., Kamishina, H., Mizukami, K., Momoi, Y., Katayama, M., Rahman, M. M., Uddin, M. M., Yabuki, A., Kohyama, M., & Yamato, O. (2013). Genotyping Assays for the Canine Degenerative Myelopathy-Associated c.118G>A (p.E40K) Mutation of the *SOD1* Gene Using Conventional and Real-Time PCR Methods: A High Prevalence in the Pembroke Welsh Corgi Breed in Japan. *Journal of Veterinary Medical Science*, *75*(6), 795–798. https://doi.org/10.1292/jvms.12-0451

Kountourantzis, A., Minoudi, S., Karaiskou, N., Papakostas, S., Moulistanos, A., Baka, R. D., Tsartsianidou, V., Vlachavas, A., Aivaliotis, M., Polizopoulou, Z. S., & Triantafyllidis, A. (2023). Prevalence of SOD1 allele associated with degenerative myelopathy in canine population in Greece. *Research in Veterinary Science*, *162*, 104959. https://doi.org/10.1016/j.rvsc.2023.104959

Santos, C. R. O., Gouveia, J. J. D. S., Gouveia, G. V., Bezerra, F. C. M., Nogueira, J. F., & Baraúna Júnior, D. (2020). Molecular screening for the mutation associated with canine degenerative myelopathy (*SOD1*:c.118G > A) in German Shepherd dogs in Brazil. *PLOS ONE*, *15*(11), e0242347. https://doi.org/10.1371/journal.pone.0242347

Turba, M. E., Loechel, R., Rombolà, E., Gandini, G., & Gentilini, F. (2017). Evidence of a genomic insertion in intron 2 of *SOD1* causing allelic drop-out during routine diagnostic testing for canine degenerative myelopathy. *Animal Genetics*, *48*(3), 365–368. https://doi.org/10.1111/age.12525

Wininger, F. A., Zeng, R., Johnson, G. S., Katz, M. L., Johnson, G. C., Bush, W. W., Jarboe, J. M., & Coates, J. R. (2011). Degenerative Myelopathy in a Bernese Mountain Dog with a Novel *SOD1* Missense Mutation. *Journal of Veterinary Internal Medicine*, *25*(5), 1166–1170. https://doi.org/10.1111/j.1939-1676.2011.0760.x

Zeng, R., Coates, J. R., Johnson, G. C., Hansen, L., Awano, T., Kolicheski, A., Ivansson, E., Perloski, M., Lindblad-Toh, K., O'Brien, D. P., Guo, J., Katz, M. L., & Johnson, G. S. (2014). Breed Distribution of *SOD 1* Alleles Previously Associated with Canine Degenerative Myelopathy. *Journal of Veterinary Internal Medicine*, *28*(2), 515–521. https://doi.org/10.1111/jvim.12317
